# Low-dose corticomedullary phase CT urography with artificial intelligence iterative reconstruction for bladder cancer evaluation

**DOI:** 10.1093/bjr/tqaf315

**Published:** 2025-12-24

**Authors:** Haifeng Liu, Xinrui Jiang, Tiantian Wang, Guozhi Zhang, Ming Zhao, Wei Xiang, Hongfen Peng

**Affiliations:** Department of Radiology, Wuhan No.1 Hospital, Wuhan, Hubei 430022, China; School of Laboratory Medicine, Hubei University of Chinese Medicine, Wuhan, Hubei 430065, China; United Imaging Healthcare, Shanghai 201800, China; United Imaging Healthcare, Shanghai 201800, China; Department of Radiology, Wuhan No.1 Hospital, Wuhan, Hubei 430022, China; Department of Urology, Wuhan No.1 Hospital, Wuhan, Hubei 430022, China; Department of Radiology, Wuhan No.1 Hospital, Wuhan, Hubei 430022, China

**Keywords:** CT urography, bladder cancer, radiation dosage, artificial intelligence iterative reconstruction

## Abstract

**Objectives:**

To test the feasibility and quantify the performance of low-dose CT urography (CTU) with artificial intelligence iterative reconstruction (AIIR) for bladder cancer (BC) evaluation.

**Methods:**

A total of 122 patients undergoing CTU examination were prospectively enrolled, where the routine-dose scan (120 kVp, ref 100 mAs) at corticomedullary phase (CMP) was followed immediately by a low-dose scan (120 kVp, ref 20 mAs). Routine-dose images were reconstructed with hybrid iterative reconstruction (HIR, RD-HIR), while low-dose images were with AIIR (LD-AIIR) and HIR (LD-HIR). The image quality was first evaluated regarding streak artefacts around the bladder and then in contrast-to-noise ratio (CNR) for various manifestations of bladder wall. The diagnostic performance of BC was characterized using receiver operating characteristic (ROC) analysis, in respect to the clinical diagnostic report.

**Results:**

The effective dose at low-dose CMP was 80.2% lower than routine-dose scan (7.6 ± 1.2 vs 1.5 ± 0.3 mSv). Nineteen cases in LD-HIR were deemed clinically unacceptable for presenting severe artefacts around the bladder, while found well above the basic requirement in LD-AIIR. The highest CNR was found in LD-AIIR in all scenarios (all *P *< .001). The area under ROC curve in LD-AIIR was comparable to RD-HIR (0.988 vs 0.990, *P *= .172) and significantly higher than LD-HIR (0.988 vs 0.831, *P *< .001).

**Conclusions:**

The low-dose AIIR protocol allows for a profound dose reduction (80.2%) while maintaining reliable diagnosis of BC on corticomedullary phase CTU images.

**Advances in knowledge:**

Corticomedullary phase CTU with AIIR permits 80.2% dose reduction while preserving reliable BC diagnosis.

## Introduction

Bladder cancer (BC) is one of the most common malignant tumours of the urinary system, ranking ninth in incidence among all cancers and accounting for about 30 000 deaths all over the world.^1^^,^^2^ Cystoscopy and CT urography (CTU) are both adopted for routine diagnosis of BC, where the latter is a non-invasive method with nearly the same accuracy as compared to the former (CTU: 87% sensitivity, 99% specificity; cystoscopy: 87% sensitivity, 100% specificity).^3^ As such, CTU, implemented with either a single bolus protocol including the separate corticomedullary phase (CMP), nephrographic phase (NP), and excretory phase (EP), or a split-bolus protocol combining the NP and EP, is becoming the first-line imaging modality for evaluation of BC as well as for many other abnormalities of the urinary system.^4^^,^^5^ Over the years, the EP has been considered the phase of choice for diagnostic CT imaging of the bladder.^6–8^ One study, however, reported that CMP has the highest sensitivity (95%) and negative predictive values (99%) for BC detection in 106 patients so this phase should be used instead.^9^ In another study, it was also suggested that the detectability of small or “en plaque” urothelial lesions is higher at CMP or NP than at EP.^5^

For CTU requires multi-phase scans with long coverage of the abdominopelvic region, continuous efforts have been made in regard of dose reduction, where the obtained evidence also points out directions for future investigation.^10–16^ Hwang et al tested a low-concentration contrast media (240 mg I/mL) and low tube voltage (80 kVp) CTU protocol on 32 cases for the EP, where the objective image quality and subjective diagnostic acceptability were found in a general sense comparable to that of the conventional protocol. Information relating to BC, however, was not explicitly given.^14^ Juri et al reported that the EP images acquired at 30% dose and with iterative reconstruction (IR) were not inferior to routine-dose images with filtered back projection reconstruction (FBP) in terms of BC detection, where only the tumours with a considerable size, ranging from 5.7 up to 101.3 mm, were accounted for.^15^ Cheng et al demonstrated for the first time that the use of deep-learning based image reconstruction (DLR) was able to deliver comparable image quality with >70% dose reduction in the EP of CTU, while the impact of DLR on BC diagnosis remains speculative.^16^ Moreover, these studies, as many others seeking to test low-dose CTU, only focused on the EP.

Hybrid iterative reconstruction (HIR) is currently the routine reconstruction technique in clinical practice and has demonstrated superior performance to the classical FBP in a variety of applications.^17^ However, it is associated with a degradation of spatial resolution and variable degrees of altered image texture, which might not sufficient for low-contrast tasks.^18^ This performance gap has been partly addressed by the model-based iterative reconstruction (MBIR), a more advanced and complex version of IR algorithms, yet the high computational requirement and long reconstruction time have limited its widespread clinical application.^19^ In recent years, with the rapid development of artificial intelligence (AI), DLR has become increasingly popular, demonstrating a significant potential of obtaining high-quality images from low-dose CT scans faster than MBIR. The latest generation of DLR algorithms, namely artificial intelligence iterative reconstruction (AIIR, United Imaging Healthcare, Shanghai, China), has shown promises in a variety of low-dose CT applications, including lung screening,^20^ liver CT,^21^ paediatric chest CT,^22^ and aortic CTA.^23^^,^^24^ Yang et al found it feasible to use an ultra-low-dose CT protocol (0.18 mSv) with AIIR for CAD-based screening of pulmonary nodule.^20^ You et al reported that AIIR allowed up to 60% dose reduction for focal hepatic lesion detection as compared to the routine protocol.^21^ Zhang et al demonstrated that AIIR has the potential for significant dose reduction in chest CT (∼0.1 mSv) of patients below 3 years old.^22^ Guo et al^23^ and Shao et al^24^ tested the performance of low-dose body CTA with AIIR as part of coronary-craniocervical CTA and TAVI planning CT, respectively. It was hypothesized, thereby, in this study that AIIR might be employed for efficient BC diagnosis with low-dose CTU at the CMP.

Hence, in this prospective study, we enrolled a cohort of patients who underwent a routine-dose scan at CMP followed immediately by a low-dose scan, where the low-dose CMP images were reconstructed with AIIR. Side-by-side comparison was made in terms of the diagnostic performance for BC as well as the image quality, against the currently applied HIR.

## Methods

### Study design

This prospective study was approved by the Medical Ethics Committee of our institution. Written informed consent was obtained from all participants, who were made aware of the additional radiation dose that was <5% to that of the entire CTU.

**Figure 1.**
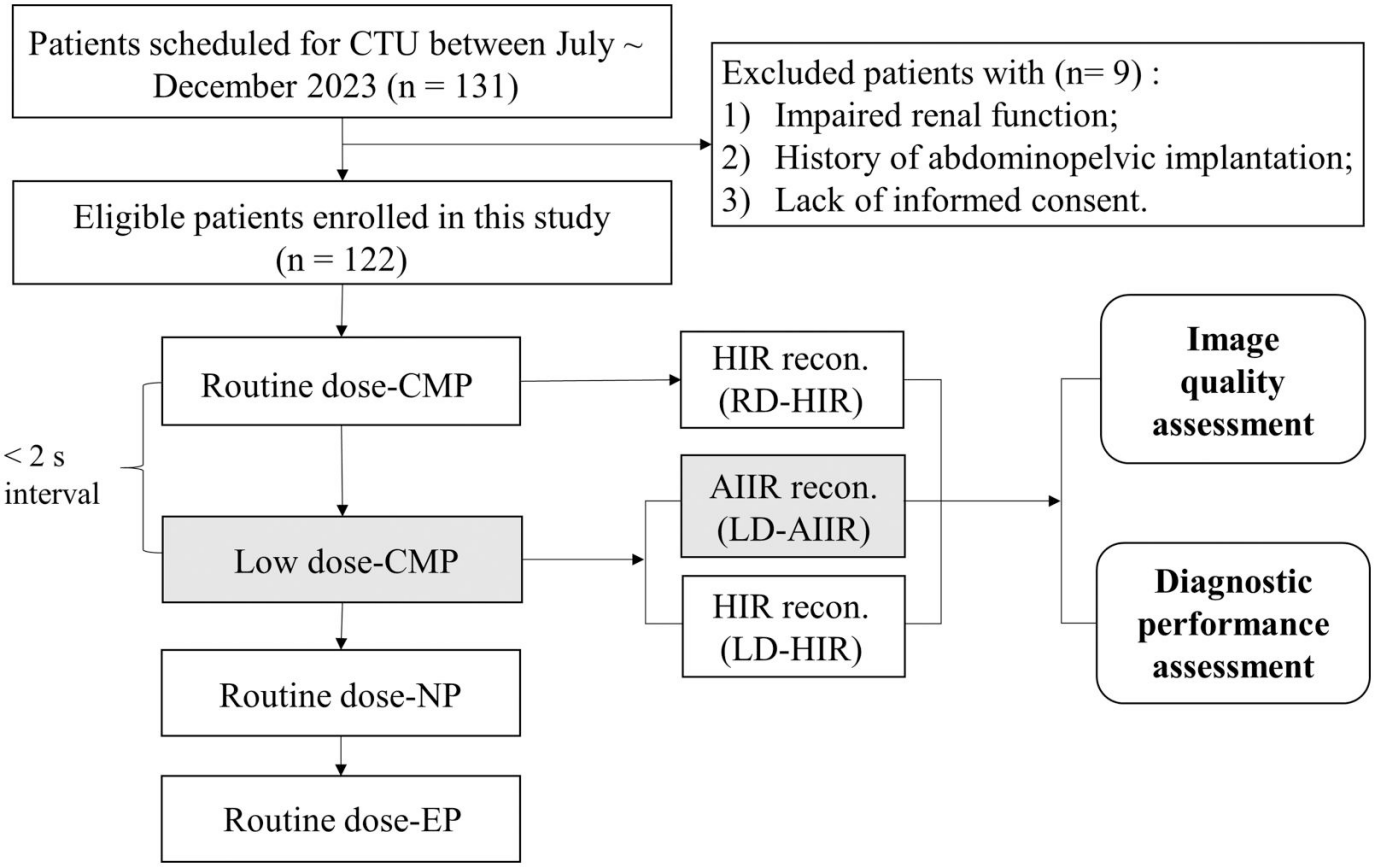
Flowchart of the study design. Abbreviations: AIIR = artificial intelligence iterative reconstruction; CMP = corticomedullary phase; CTU = CT urography; EP = excretory phase; HIR = hybrid iterative reconstruction; NP = nephrographic phase.

Figure 1 shows the flowchart of study design and case collection. From July to December 2023, a total of 131 consecutive patients with clinical indications requiring a CTU examination were considered for inclusion in this study. The exclusion criteria were: (1) impaired renal function or allergies to iodinated contrast agent where CTU was not feasible; (2) known history of metal femoral head replacement that might complicate the analysis of resulting image quality; or (3) lack of patient’s consent. A total of 122 patients (male/female: 86/36; mean age: 66.9 ± 10.3 years, range 34-90 years; mean BMI: 24.6 ± 3.7, range 14.1-37.5 kg/m^2^) were enrolled for the final analysis.

Owing to the paired image acquisition at CMP, side-by-side comparison was enabled not only between 2 different reconstructions of the low-dose data but between data acquired using 2 different dose settings on the same subject. The objective image quality assessment was carried out in such a scheme, taking into account 3 practical scenarios relating to the exhibition of bladder: (1) cases suffering from severe streak artefacts on low-dose images of either reconstruction, where the bladder wall was not sufficiently identifiable for any region of interest (ROI) measurement; (2) those presenting near the middle level a bladder wall of ≥3 mm in thickness, either on average or partially, which would allow for a ROI to be placed for measurement; and (3) those with a bladder wall of <3 mm in thickness that the measurement had to rely on ROIs placed in the lumen or on adjacent organs group. The difference of bladder wall, as between (2) and (3), could be a result of the filling state of the bladder at the time of imaging or a pathological indication. Diagnostic performance, on the other hand, was characterized using all cases and images.

### CTU image acquisition

All CTU scans were performed on an 80-row CT scanner (uCT 780, United Imaging Healthcare, Shanghai, China) with the following acquisition parameters: scan mode, helical; tube voltage, 120 kVp; rotation time, 0.5 s; longitudinal collimation, 80 mm; auto-modulated tube current, with a reference of 100 mAs for the routine-dose scan and of 20 mAs for the low-dose scan, aiming for an 80% dose reduction. It is worth mentioning that the routine-dose scan was acquired in craniocaudal direction and then the low-dose CMP scan followed immediately in caudalcranial direction, where the gap was reduced to <2 s, allowing for the minimal discrepancy of the 2 scans in terms of the contrast enhancement.

Iodinated contrast medium (Iomeprol 400 mgI/mL, Bracco Imaging Italia S.r.L.) was intravenously injected using a weight-dependent volume of 1.2 ml/kg with a flow rate of 3.0 mL/s, followed by 30 mL of saline solution at the same rate. The ROI for bolus tracking was configured on the descending aorta with a triggering threshold of 120 Hounsfield Units (HU). The routine-dose CMP, low-dose CMP, routine-dose NP, and routine-dose EP images were acquired at 15 s, 22.5 s, 70 s, and 300 s after the scan was triggered, respectively, where the delay of CMP from the beginning of injection was 30-45 s depending on the patient’s cardiovascular function.

All CMP scans were reconstructed with a slice thickness/increment of 1.0/1.0 mm and a matrix size of 512 × 512 pixels, respectively. The routine-dose CMP images were reconstructed with HIR (Karl 3D, United Imaging Healthcare, Shanghai, China), referred to as RD-HIR, while the low-dose CMP images were reconstructed with AIIR (LD-AIIR) and HIR (LD-HIR), resulting in a total of 366 image sets (122 patients × 3 reconstructions) for further analysis. Technically, AIIR is featured by replacing the traditional regularization term in MBIR for noise suppression with a dedicated de-noising convolutional neural network (CNN), therefore combing the advantage of MBIR in characterizing image details with the strength of CNN in effectively handling image noise and texture.^25^ The AIIR is trained with millions of image pairs, that is standard-dose images and the corresponding simulated low-dose images, which were considered noise-free and noise-contaminated, respectively. To improve the generalizability and robustness of AIIR, a variety of datasets for multiple body parts, scan protocols, radiation dose levels, were used for the training.

The volume CT dose index (CTDIvol, mGy) and dose length product (DLP, mGy cm) were recorded. The effective dose (ED) was calculated as DLP multiplied by the abdomen-pelvic conversion factor of 0.015 mSv mGy^−1^ cm^−1^.^26^

### Image quality assessment

In the first step of the assessment, 2 diagnostic radiologists (with 12 and 14 years of experience in abdominal radiology each), blinded to the scanning protocols, reconstruction algorithms, and all patient information, were invited to independently review all low-dose images and select those with severe streak artefacts around the bladder that were no longer acceptable for clinical diagnosis. The low-dose images, reconstructed by HIR or AIIR, were mixed and randomly presented on the routine clinical workstation (uWS-CT, United Imaging Healthcare, Shanghai, China). Consensus was achieved through negotiation in case of disagreement between the 2 readers.

In the second step, a third radiologist, with 6 years of experience in radiology assessed the objective image quality of 3 image sets, in terms of the contrast-to-noise ratio (CNR) relating to the bladder, for all remaining cases. As mentioned above, for patients with a bladder wall ≥3 mm, the CNR was defined between the bladder wall and the lumen (CNR_wall to lumen_). For patients with a bladder wall <3 mm, the CNR was defined between the lumen and one adjacent organ, which was the prostate for male patients and uterus for the female, that is CNR_prostate to lumen_ and CNR_uterus to lumen_. The measurement was performed using circular ROIs for 3 times and averaged, where the sizes were: (1) 200-300 mm^2^ on the lumen, depending on the filling state of the bladder; (2) 30-50 mm^2^ on the bladder wall, depending on the wall thickness; and (3) 50-100 mm^2^ on the adjacent organs (prostate/uterus). The ROIs were carefully placed on routine-dose images while avoiding the lesions, and then copied-pasted onto the corresponding low-dose images. The CNR was calculated for the wall or the adjacent organ against the lumen with:


(1) $$\mathrm{CNR}=\frac{\left|\mathrm{HU}-{\mathrm{HU}}_{\mathrm{lumen}}\right|}{{\mathrm{SD}}_{\mathrm{lumen}}}$$

where HU and SD denote the mean CT value and the standard deviation, respectively, in Hounsfield Units.

### Diagnostic performance assessment

The 2 radiologists who performed the first step imaging quality assessment were invited to grade the confidence in diagnosing BC on RD-HIR, LD-AIIR, and LD-HIR images, using a 5-point scale: 1, definitely absent; 2, likely absent; 3, indeterminate; 4, likely present; 5, definitely present. This diagnostic performance assessment was performed after a washout period of at least 1 month from the initial image quality assessment to minimize recall bias. Each radiologist evaluated RD-HIR, LD-AIIR, and LD-HIR images, including those found with severe streak artefacts in 3 separate sessions, with an interval period of 2-3 weeks to reduce recall bias. For each session, images were presented in a random order and radiologists were instructed to read in a manner similar to clinical practice. Multi-planar reconstruction (MPR) images were produced each time by the readers as needed. Consensus was again obtained through negotiation in case of disagreement.

The reference standard about BC was established according to the clinical diagnostic report of each patient, including pathological results of the cases that were considered necessary (cystoscopy and biopsy) and call clinical data that were available at the time of the diagnosis. The diagnostic performance for BC detection among 3 image sets was able to be evaluated via a receiver operating characteristic (ROC) analysis by varying the threshold of confidence score (1-5).

### Statistical analysis

All statistical analyses were performed using SPSS version 27 (IBM Corp., Armonk, NY, United States). Continuous variables were presented as mean ± standard deviation (SD) or median and interquartile range (IQR) as appropriate. The Kolmogorov-Smirnov test was used to examine the normality of continuous variables. For data with normal distribution, the Student’s *t*-test for paired samples was used, otherwise the non-parametric Wilcoxon signed-rank test was used. The area under the ROC curve (AUC) with 95% confidence interval (CI) was employed to compare the resulting diagnostic performance, while the sensitivity, specificity and accuracy were calculated based on a threshold of score 3. A 2-tailed *P *< .05 was considered statistically significant.

## Results

### Patient characteristics

The patient characteristics are listed in Table 1. The majority of patients underwent CTU due to haematuria (41.8%). In addition to CTU, 55 patients (45.1%) were referred to further pathological exams by cystoscopy and biopsy within 1 week. Among 122 patients, 46 (37.7%) were confirmed with BC according to the reference standard.

**Table 1.** Patient characteristics.

Parameter	Number/value
Number of patients	122
Age (years)	
Average ± SD	66.9 ± 10.3
Range	34-90
Sex, *n* (%)	
Female	36 (29.5%)
Male	86 (70.5%)
BMI (kg/m^2^)	
Average ± SD	24.6 ± 3.7
Range	14.1-37.5
Clinical indication, *n* (%)	
Haematuria	51 (41.8%)
Abdominal pain	10 (8.2%)
Voiding dysfunction	6 (4.9%)
Pre-operative assessment	5 (4.1%)
Re-examination of cancer	18 (14.8%)
Follow-up of abnormalities in urinary system	25 (20.5%)
Hypertension or anaemia	5 (4.1%)
Others	2 (1.6%)
Other examinations, *n* (%)	
Cystoscopy and biopsy	55 (45.1%)
Urine cytology	43 (35.3%)
Diagnostic finding, *n* (%)	
Bladder cancer	46 (37.7%)
Renal cancer	13 (10.7%)
Adrenal tumour	5 (4.1%)
Prostate cancer	3 (2.5%)
Hysteromyoma	2 (1.6%)
Renal cyst	20 (16.4%)
Urinary calculi	6 (4.9%)
Hydronephrosis with urinary calculi	13 (10.7%)
Prostatic hyperplasia	7 (5.7%)
Cystitis	5 (4.1%)
Others	2 (1.6%)

For the routine-dose CMP images, the mean CTDIvol, DLP, and ED were 11.1 ± 1.4 mGy, 506.1 ± 82.5 mGy cm, and 7.6 ± 1.2 mSv, respectively. For the low-dose CMP images, that was 2.2 ± 0.3 mGy, 100.0 ± 16.5 mGy cm, and 1.5 ± 0.3 mSv, respectively, showing up to 80.2% dose reduction.

### Image quality

**Figure 2.**
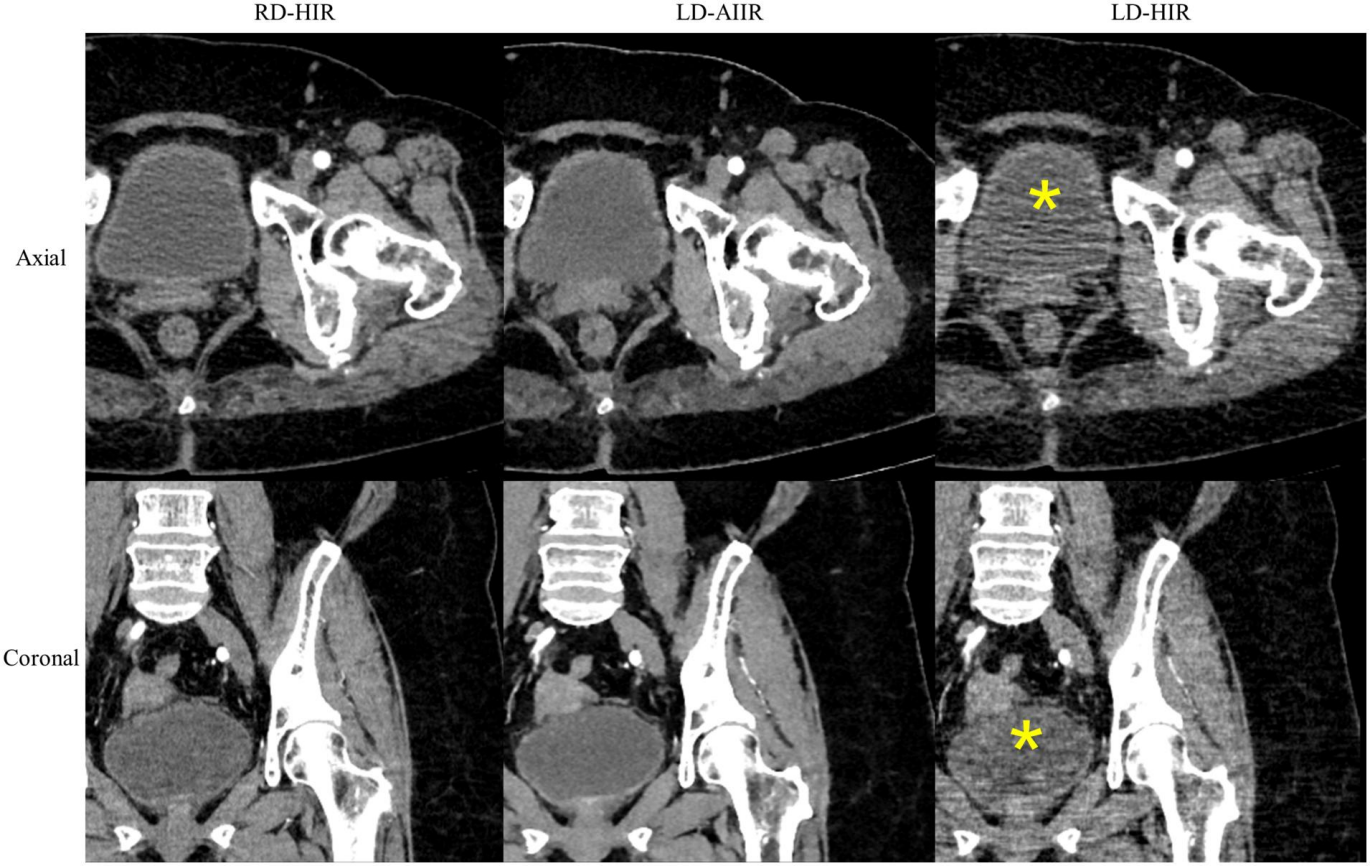
Corticomedullary phase CT images of 63-year-old woman at routine-dose with hybrid iterative reconstruction (RD-HIR), low-dose with artificial intelligence iterative reconstruction (LD-AIIR) and with hybrid iterative reconstruction (LD-HIR), where LD-HIR images failed to meet clinical requirements due to severe streak artefacts around the bladder (yellow *).

Figure 2 shows a case where excessive streak artefacts are present on the LD-HIR images. A total of 19 (15.6%) such cases were identified and deemed clinically unacceptable in consensus opinion, on which further quantitative measurement regarding the bladder was infeasible or no longer necessary. The paired AIIR images at the same low dose, on the contrary, were all found well above the basic diagnostic requirement, with only mild or negligible artefacts.

**Figure 3.**
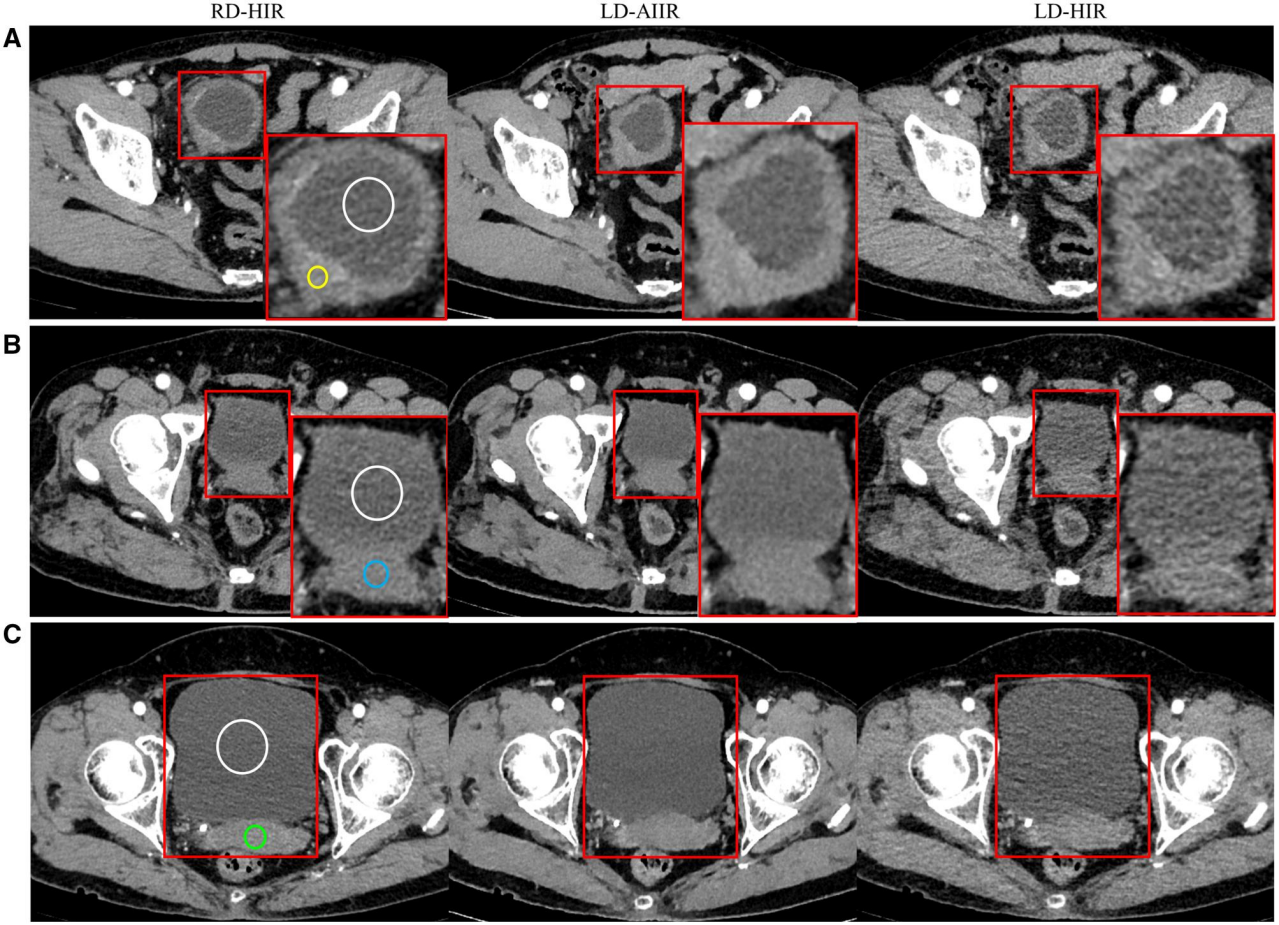
Corticomedullary phase CT images of (A) a 69-year-old man, (B) a 55-year-old man and (C) a 58-year-old woman at routine-dose with hybrid iterative reconstruction (RD-HIR), low-dose with artificial intelligence iterative reconstruction (LD-AIIR)and with hybrid iterative reconstruction (LD-HIR), where the contrast-to-noise ratio is shown for the (A) bladder wall (yellow) and lumen (white), (B) bladder lumen (white) and prostate (blue), and (C) bladder lumen (white) and uterus (green).

Then, the rest 103 cases were considered in the second step of assessment, including 52 cases with bladder wall ≥3 mm and 51 cases with bladder wall <3 mm. As shown in Figure 3, the AIIR reconstruction managed to suppress the noise at low radiation dose and provided a complete, conspicuous depiction of the bladder. Although the magnitude of the noise appears to be even less prominent than that on the RD-HIR images, no signs of detailing being smeared, as usually seen in cases of excessive de-noising, were noticed on the LD-AIIR images. On the contrary, various small detects are found around the bladder on the LD-HIR images, suggesting an insufficient exposure setting.

The CNR relating to the bladder on the 3 image sets is present in Table 2. The CNR_wall to lumen_ for those with a bladder wall ≥3 mm, the CNR_prostate to lumen_ for male patients with a bladder wall <3 mm, and the CNR_uterus to lumen_ for female patients with a bladder wall <3 mm were all significantly higher on LD-AIIR images compared to that on LD- and RD-HIR images, where *P* from the Student’s *t*-test for pairwise comparison were all <0.001. Worth mentioning is that, as indicated by the results on RD-HIR images, the CNR_wall to lumen_ for those with a bladder wall ≥3 mm seems to be comparable to the CNR_uterus to lumen_ for female patients showing a <3 mm bladder wall on the image.

**Table 2.** Comparison of the contrast-to-noise ratio defined with respect to the manifestation of the bladder on images of different dose settings and reconstruction methods.

	RD-HIR	LD-AIIR	LD-HIR	*P*_1_	*P*_2_
Bladder wall ≥3 mm (*n* = 52)					
CNR_wall to lumen_	3.6 ± 1.2	5.0 ± 1.8	2.8 ± 1.0	<0.001	<0.001
Bladder wall <3 mm, male (*n* = 31)					
CNR_prostate to lumen_	2.3 ± 0.7	3.2 ± 0.9	1.8 ± 0.6	<0.001	<0.001
Bladder wall <3 mm, female (*n* = 20)					
CNR_uterus to lumen_	3.5 ± 0.7	4.6 ± 1.0	2.8 ± 0.6	<0.001	<0.001

Data are mean ± standard deviation.

Abbreviations: AIIR = artificial intelligence iterative reconstruction; HIR = hybrid iterative reconstruction; LD = low dose; *P*_1_ and *P*_2_ = *P-*value from the Student’s *t*-test for pairwise comparison between RD-HIR and LD-AIIR, and LD-AIIR and LD-HIR, respectively; RD = routine dose.

### Diagnostic performance

**Figure 4.**
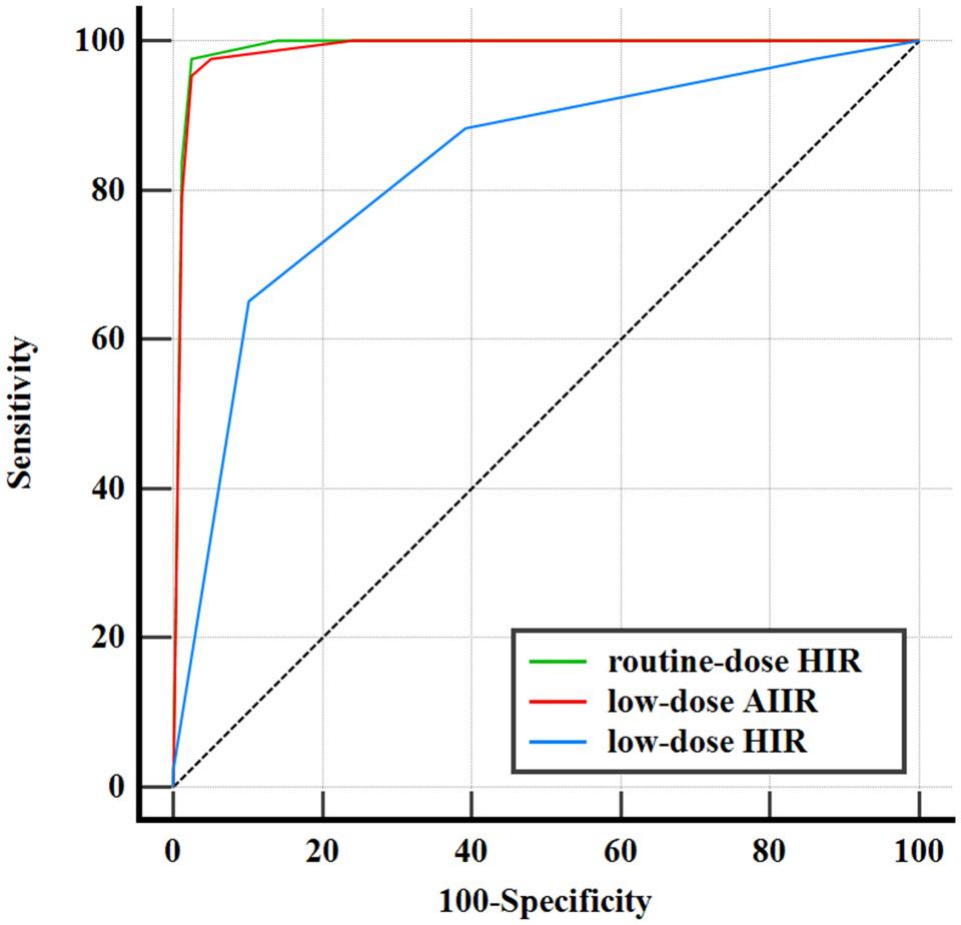
Receiver operating characteristic curves of routine-dose with hybrid iterative reconstruction (HIR), low-dose with artificial intelligence iterative reconstruction (AIIR) and with HIR images in detecting bladder cancer, where the area under curve (AUC) was 0.990 (95% CI: 0.952, 1), 0.988 (95% CI: 0.970, 1), and 0.831 (95% CI: 0.752, 0.910) for routine-dose HIR, low-dose AIIR, and low-dose HIR images, respectively.

A total of 46 (37.7%) patients were confirmed with BC according to the reference standard. Figure 4 and Table 3 show the diagnostic performance for BC on 3 image sets. The AUC of LD-AIIR and RD-HIR images was comparable (0.988 vs 0.990, *P *> .05). Similarly, the sensitivity, specificity, and accuracy for BC detection on LD-AIIR scans (97.7%, 94.9, and 95.9%, respectively) were comparable with the corresponding values on RD-HIR scans (97.7%, 97.5, and 97.5%, respectively).

**Table 3.** Comparison of the diagnostic performance for bladder cancer detection on images of different dose settings and reconstruction methods.

	RD-HIR	LD-AIIR	LD-HIR
AUC	0.990 (0.951, 1)	0.988 (0.948, 0.999)	0.831 (0.752, 0.893)
Sensitivity (%)	97.7 (87.7, 99.9) [42/43]	97.7 (87.7, 99.9) [42/43]	88.4 (74.9, 96.2) [38/43]
Specificity (%)	97.5 (91.2, 99.7) [77/79]	94.9 (87.5, 98.6) [75/79]	60.8 (49.2, 71.6) [48/79]
Accuracy (%)	97.5 (93.0, 99.5) [119/123]	95.9 (90.7, 98.7) [117/123]	70.5 (61.6, 78.4) [86/123]

Numbers in parentheses are 95% confidence intervals (CIs); numbers in brackets are the number of lesions.

Abbreviations: AIIR = artificial intelligence iterative reconstruction; AUC = area under the receiver operative characteristic curve; HIR = hybrid iterative reconstruction; LD = low dose; RD = routine dose.

**Figure 5.**
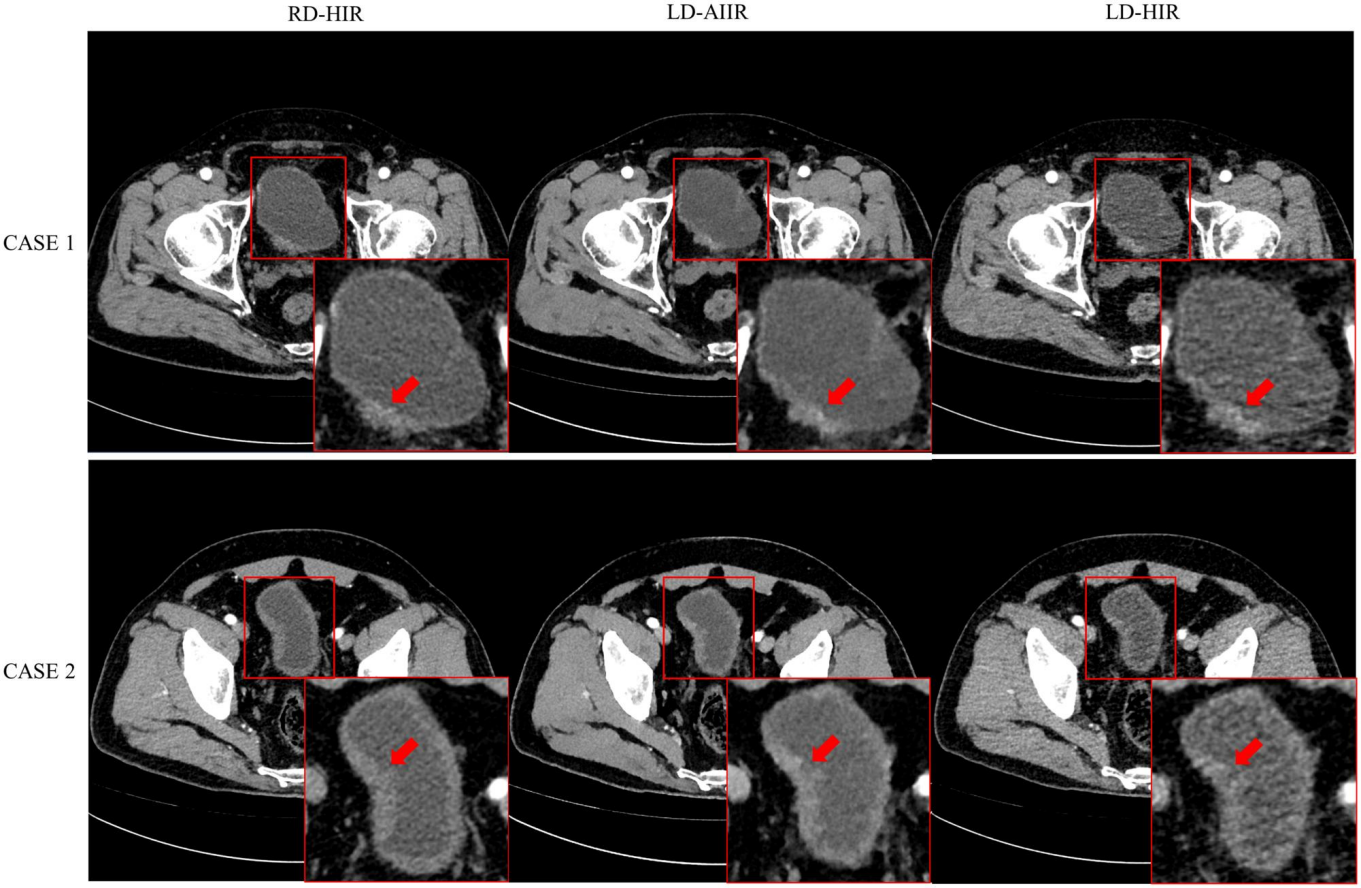
Corticomedullary phase CT images of a 72-year-old man (CASE 1) and a 60-year-old man (CASE 2) at routine-dose with hybrid iterative reconstruction (RD-HIR), low-dose with artificial intelligence iterative reconstruction (LD-AIIR), and with hybrid iterative reconstruction (LD-HIR). Pathology results confirmed both cases were negative for bladder cancer. The diagnostic confidence on RD-HIR, LD-AIIR, and LD-HIR was graded 4, 4 and 4 for CASE 1; and 2, 3, and 4 for CASE 2, respectively, by both radiologists.

Three cases (positive/negative: 1/2) were misclassified on both LD-AIIR and RD-HIR images but corrected by pathology. One positive case was missed because the lesion was <3 mm in size and without sufficient enhancement, leaving it nearly completely invisible on both image sets. This shows the limitation of CT in detecting very small or flat lesions, regardless of the dose or reconstruction algorithm. The 2 cases were false positive, one presented in Figure 5 (CASE 1) and both confirmed as benign inflammation by pathology, where the presence of wall thickening with relatively high signal intensity on the right side of the bladder was mistaken as malignant features. Two additional negative cases of false positives were found with LD-AIIR but correctly diagnosed on RD-HIR images, one of which is shown in Figure 5 (CASE 2). The key discriminator for different diagnosis was again the signal intensity of the “mass” on the wall, which appears to be abnormally high on both LD-HIR and LD-AIIR images but not on the RD-HIR image.

In addition, significant improvement was found on LD-AIIR compared to LD-HIR images (*P *= .001), with the AUC being 0.988 vs 0.831, the sensitivity being 97.7% vs 88.3%, the specificity being 94.9% vs 60.8%, and the accuracy being 95.9% vs 70.5%. A total of 33 cases that were graded indeterminate (score 3) on LD-HIR images, while were correctly diagnosed on the LD-AIIR images. Among 64 cases that were correctly diagnosed but with limited confidence (score 2/4) on the LD-HIR images, 63 were graded in the same direction and with greater confidence (score 1/5) with the AIIR.

**Figure 6.**
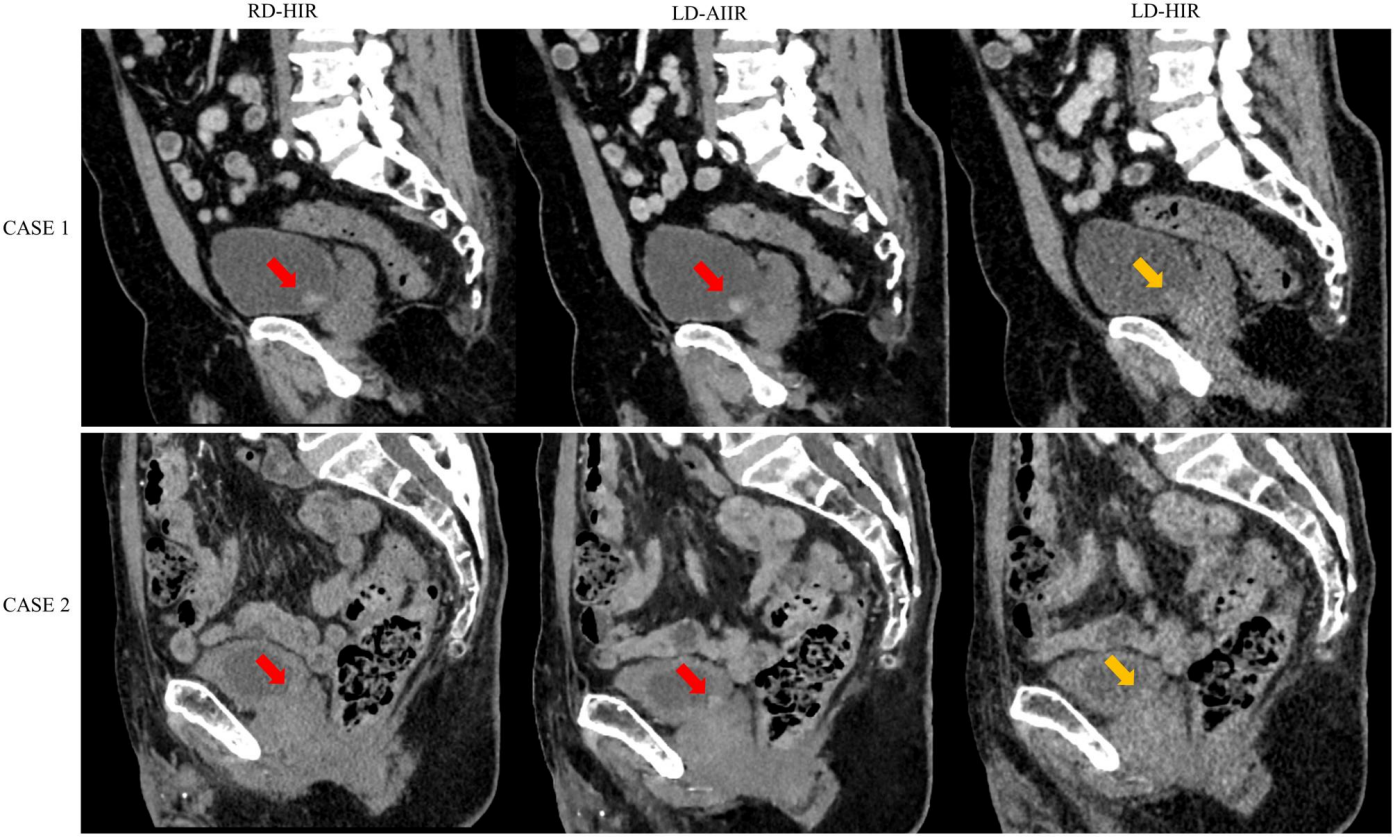
Corticomedullary phase CT images of a 75-year-old man (CASE 1) and a 87-year-old man (CASE 2) at routine-dose with hybrid iterative reconstruction (RD-HIR), low-dose with artificial intelligence iterative reconstruction (LD-AIIR), and with hybrid iterative reconstruction (LD-HIR). Pathology results confirmed both cases were positive for bladder cancer (arrow). The diagnostic confidence on RD-HIR, LD-AIIR, and LD-HIR was graded 4, 4 and 3 for CASE 1; and 4, 4, and 2 for CASE 2, respectively, by both radiologists.

Figure 6 shows a case with limited diagnostic confidence and a case of misdiagnosis on the LD-HIR images, respectively, while both cases with BC confirmed by cystoscopy and biopsy were correctly diagnosed on both RD-HIR and LD-AIIR images. When evaluating the first case, both radiologists were uncertain in the diagnosis (score 3) due to the major noise and poor contrast with the surrounding tissues in the LD-HIR image, while were able to diagnose the likely presence of BC (score 4) in the RD-HIR and LD-AIIR images, which might be attributed to the high CNR of both images that enabled an optimal contrast of bladder, increasing the conspicuity of bladder lesions. When evaluating the second case, both radiologists graded likely absent (score 2) due to the nearly invisible lesions in the LD-HIR image, while graded likely present (score 4) due to the clearly visible lesions in the RD-HIR and LD-AIIR images.

## Discussion

To the best of our knowledge, this study was the first study to evaluate the effect of DLR on diagnosing BC and improve image quality of low-dose CTU. Results revealed that low-dose AIIR images with a significantly dose reduction of 80.2% offered diagnostic acceptability comparable with routine-dose HIR images in BC, and low-dose AIIR images yielded better image quality with lower image noise and higher CNR than low-dose and routine-dose HIR images.

Optimization of radiation dose following the principle of as low as reasonably achievable (ALARA) is an ongoing topic of great concern in radiology. In previous studies, radiation dose reduction on CTU was all performed on EP or NP, with the proportion of reduction up to 71%.^10–16^ In our study, for the first time, we attempted to reduce dose on CMP by up to 80.2%. The CMP was selected for investigation because it has been reported to have the highest sensitivity (95%) and negative predictive values (99%) for BC detection and is recommended for bladder assessment.^9^ Our low-dose AIIR images yielded similar diagnostic performance, with the values of 96% and 99%, respectively. The promising results suggest that AIIR has the potential to redefine clinical CTU protocols by enabling substantial radiation dose reduction in CMP without compromising diagnostic performance.

Low-dose CTU has been explored in previous studies with a focus on the EP. In Hwang et al, where the ED of EP was 3.4 ± 1.4 mSv, the CNR between the bladder and psoas muscle was found to be 47.2 ± 36.5 by an IR algorithm, namely iDose,^4^^,^^14^ In Cheng et al, that was 62.8 ± 43.9 with an ED of 2.0 ± 0.4 mSv by using a DLR algorithm, namely TrueFidelity.^16^ To enable a direct comparison, a small additional cohort of patients (*N* = 8) was included in this study and took a low-dose EP instead of low-dose CMP, with the same setting of dose reduction. AIIR achieved a CNR of 62.3 ± 38.0 with a substantially lower ED of 1.5 ± 0.3 mSv, suggesting it may be stronger in noise suppression than the other 2 advanced reconstruction algorithms. Additionally, comparing CMP and EP, the CNR was 3.7 ± 0.7 vs 4.8 ± 1.0 on routine-dose HIR images and low-dose AIIR images, respectively, for the CMP, and 47.2 ± 31.4 vs 62.3 ± 38.0 on those, respectively, for the EP. In terms of percentage, AIIR delivered a similar CNR improvement (∼32%) for the 2 phases, where EP was with contrast media in the bladder and CMP was without contrast in cavity.

According to the routine protocol, pathological confirmation was only conducted for those with substantial clinical indication, which was the case for 55 out of 122 patients (positive/negative: 46/9). For the other 67 patients, no sign of bladder abnormalities was observed at all, so no pathological confirmation was required and these cases were considered negative for BC. However, it is worth mentioning that 19 out of these 67 patients did undergo pathological examination for indications other than BC, where it has helped determine adrenal tumour (*n* = 2), renal tumour (*n* = 9), renal cyst (*n* = 2), ureter tumour (*n* = 1), ureteral inflammation (*n* = 1), and prostatic hyperplasia (*n* = 4). From another perspective, pathological confirmation could also be of interest in interpreting the results of the present investigation. As suggested by the grading of diagnostic confidence, it becomes more challenging to rule out BC with HIR images at reduced dose, which might have led to more patients being referred to pathological examination than with AIIR.

This study has some limitations that need to be considered. First, the AIIR algorithm is vendor-specific, for which the extrapolation of the current findings needed to be cautious and the reproducibility remained of interest to further explorations. However, the demonstrated investigative design can be easily translational to other DLR algorithms and vendors’ CT scanners. Second, although a blinded reading design was employed for the evaluation, potential biases due to the inherent difference in image appearance between 2 scanning protocols and algorithms remained inevitable and should be considered when interpreting the results. Last, neither patient follow-up information nor images of other modalities, such as multiparametric MR in combination with AI that has been recently reported feasible in diagnosing and monitoring BC,^27^ were available for comparing with the findings in this investigation, which would have allowed for a more comprehensive assessment of the reliability of low-dose CTU.

In conclusion, the AIIR algorithm enables up to 80.2% dose reduction in CTU imaging of the BC. Its demonstrated capacity in dose reduction and the superior imaging performance over the current routine HIR suggest great potential for adoption in various routine CTU applications. Following the present study, extending the use of AIIR to other phases or the entire exam of CTU for dose reduction and for clinical indications other than BC is attractive and worthy of exploration.
